# ^1^H high resolution magic-angle coil spinning (HR-MACS) μNMR metabolic profiling of whole *Saccharomyces cervisiae* cells: a demonstrative study

**DOI:** 10.3389/fchem.2014.00038

**Published:** 2014-06-12

**Authors:** Alan Wong, Céline Boutin, Pedro M. Aguiar

**Affiliations:** ^1^CEA Saclay, DSM, IRAMIS, UMR CEA/CNRS 3299 – NIMBE, Laboratoire Structure et Dynamique par Résonance MagnétiqueGif-sur-Yvette, France; ^2^Department of Chemistry, University of YorkHeslington, York, UK

**Keywords:** micro-NMR, HR-MACS, Metabolic Profiling, *Saccharomyces cervisiae*, osmotic stress, cell growth

## Abstract

The low sensitivity and thus need for large sample volume is one of the major drawbacks of Nuclear Magnetic Resonance (NMR) spectroscopy. This is especially problematic for performing rich metabolic profiling of scarce samples such as whole cells or living organisms. This study evaluates a ^1^H HR-MAS approach for metabolic profiling of small volumes (250 nl) of whole cells. We have applied an emerging micro-NMR technology, high-resolution magic-angle coil spinning (HR-MACS), to study whole *Saccharomyces cervisiae* cells. We find that high-resolution high-sensitivity spectra can be obtained with only 19 million cells and, as a demonstration of the metabolic profiling potential, we perform two independent metabolomics studies identifying the significant metabolites associated with osmotic stress and aging.

## Introduction

^1^H nuclear magnetic resonance (NMR) spectroscopy has gained recognition as a key analytical technique for metabolic profiling of complex bio-systems (Dunn and Ellis, 2005). The non-destructive nature, simplicity of sample preparation and data acquisition provide advantages over other methods including mass spectrometry (MS) for high-precision investigation of metabolites in living specimens. Moreover, the observed signal intensities in a typical ^1^H NMR spectrum provide a *direct* comparison of the metabolite contents in the samples without the need to construct calibration curves for every analyte, which is often the case for other analytical techniques. For these reasons, ^1^H NMR spectroscopy is widely used in the study of metabolomes, offering a robust tool for rich-metabolic profiling (Reo, 2002). Many samples of interest are highly complex bio-mixtures (e.g., biopsies, whole living cells and organisms) and the heterogeneity in the magnetic susceptibility over the sample volume results in broadening of the observed NMR resonances; significantly reducing the ability to identify and quantify the metabolic content using traditional high-resolution NMR techniques. For such samples the application of ^1^H-detected high-resolution magic-angle spinning (HR-MAS) NMR, has now emerged as a powerful analytical tool for the investigation of heterogenous samples such as intact biopsies (Lindon et al., 2009; Sitter et al., 2009; Beckonert et al., 2010) and whole living organisms (Blaise et al., 2007; Righi et al., 2010). The rapid spinning of the sample (ca. 2–6 kHz) about an axis at an angle of 54.74° (i.e., the magic-angle) with respect to the static magnetic field *B*_0_ overcomes the broadening and yields well-resolved signals. ^1^H HR-MAS NMR is considered a near universal technique for providing unbiased and high-precision fingerprints of abundant metabolites in heterogenous biosamples. Despite its utility, there are only a handful of studies on whole cells, mainly with robust eukaryotic cells such as marine unicellular microalgae cells (Chauton et al., 2003a,b) and bacterial cells (Himmelreich et al., 2003; Gudlavalleti et al., 2006; Palomino-Schätzlein et al., 2013; Righi et al., 2013), whose cell membranes provide additional protection and structural support significantly enhancing cell survival rates during the measurement. In a review by Li (2006), he has summarized a progress for *in vivo* studies of intact bacterial cell using multidimensional NMR experiments with HR-MAS. This paucity of examples for HR-MAS studies of whole cells, compared to tissues, is due in large part to the potential for cell lysis as a result of the large centrifugal forces that the cells are subject to under the rapid sample spinning. This is especially problematic for fragile animal and human cells, and can result in distortion of the intracellular metabolic composition. Thus, high-resolution NMR of cell extracts has been the preferred method for cell studies. However in this case, the cells must be subject to complicated chemical treatment protocols to extract specific metabolites; hydrophilic metabolites in aqueous extracts and lipophilic in organic extracts, and require larger sample quantities for the multiple NMR samples and experiments to obtain a rich profile of the metabolic response. Unlike with the analysis of extracts, it is unnecessary to chemically divide the hydrophilic and hydrophobic metabolites for HR-MAS, offering a direct analytical approach. Palomino-Schätzlein et al. have optimized a HR-MAS protocol for the study of whole cells using abundant *Saccharomyces cervisiae* cells and reported similar metabolic profiles to those obtained with high-resolution NMR of cell extracts (Palomino-Schätzlein et al., 2013).

NMR is an inherently insensitive technique, thus HR-MAS analysis often relies on large sample volumes for detection; typically about 100 million whole cells in a 30–50 μl volume for each of 3–5 replicate samples (for statistical analyses). In cases where sample size is limited (such as neuron cells), analysis of fewer cells—in a smaller volume—would ease the sample preparation and may improve the high-throughput efficiency (e.g., coupling with micro-fluidic devices for cell separation techniques). One promising approach for volume-, or mass-, limited bio-specimens is the uses of a high-resolution magic-angle coil spinning (HR-MACS) (Wong et al., 2012, 2013). HR-MACS, as with the original MACS experiment (Sakellariou et al., 2007), utilizes a secondary tuned circuit and a simple and robust rotor insert, designed to fit inside a standard MAS sample rotor to convert the MAS probe into a μMAS probe without any probe modification. HR-MACS can be readily coupled with any standard HR-MAS probe making it assessable to any laboratory with HR-MAS facilities. The use of a filling-factor optimized detector, susceptibility-optimized inserts and simultaneous spinning of the sample and detector have been demonstrated to yield high sensitivity and excellent spectral resolution (up to 2 ppb) allowing for high-precision metabolomic assessments of a sample volume less than 500 nl (Wong et al., 2013). Moreover, the reduced diameter of the sample also abates the centrifugal forces exerted on the cells under sample spinning diminishing the chances of cell lysis due to sample spinning.

The present study capitalizes on the new development of HR-MACS for high-sensitivity and high-resolution ^1^H μNMR-based metabolomics study of whole cells. We build upon the ^1^H HR-MAS study of whole *S. cervisiae* cells (Palomino-Schätzlein et al., 2013) to evaluate the utility of ^1^H HR-MACS for profiling the metabolic composition of a small number of *S. cervisiae* cells (ca. 19 million cells in a 250 nl volume). We have performed two independent studies, monitoring the metabolic responses in *S. cervisiae* wild-type cells under osmotic stress and cell growth. There are a total of four cell groups submitted to the two studies: 24, 48, and 72-h cell growth and a 24-h cell growth subjected to a 60-min period of osmotic stress.

## Materials and methods

### Saccharomyces cervisiae

Wild-type (WT) cells were grown in a glucose-rich medium YPD (1% yeast extract, 2% peptone, 2% glucose) at 30°C on a shaker (180 rpm). Each sample was inoculated with the same initial culture and with an initial OD_600_ of 0.1 and grown for 24, 48, and 72 h. For the osmotic cell stress study, 0.5 M NaCl (final concentration) was added to the 24-h culture cells for 60 min. Prior to the NMR sample preparation, *ca*. 300 × 10^6^ cells were washed 3 times with ion-free distilled water. The cell pellet was re-suspended in 4 μl of D_2_O for an immediate NMR acquisition. The use of high cell concentration was utilized to ensure significant numbers of cells were being transferred to the microsize capillary.

### HR-MACS resonator

**Figure 1:**
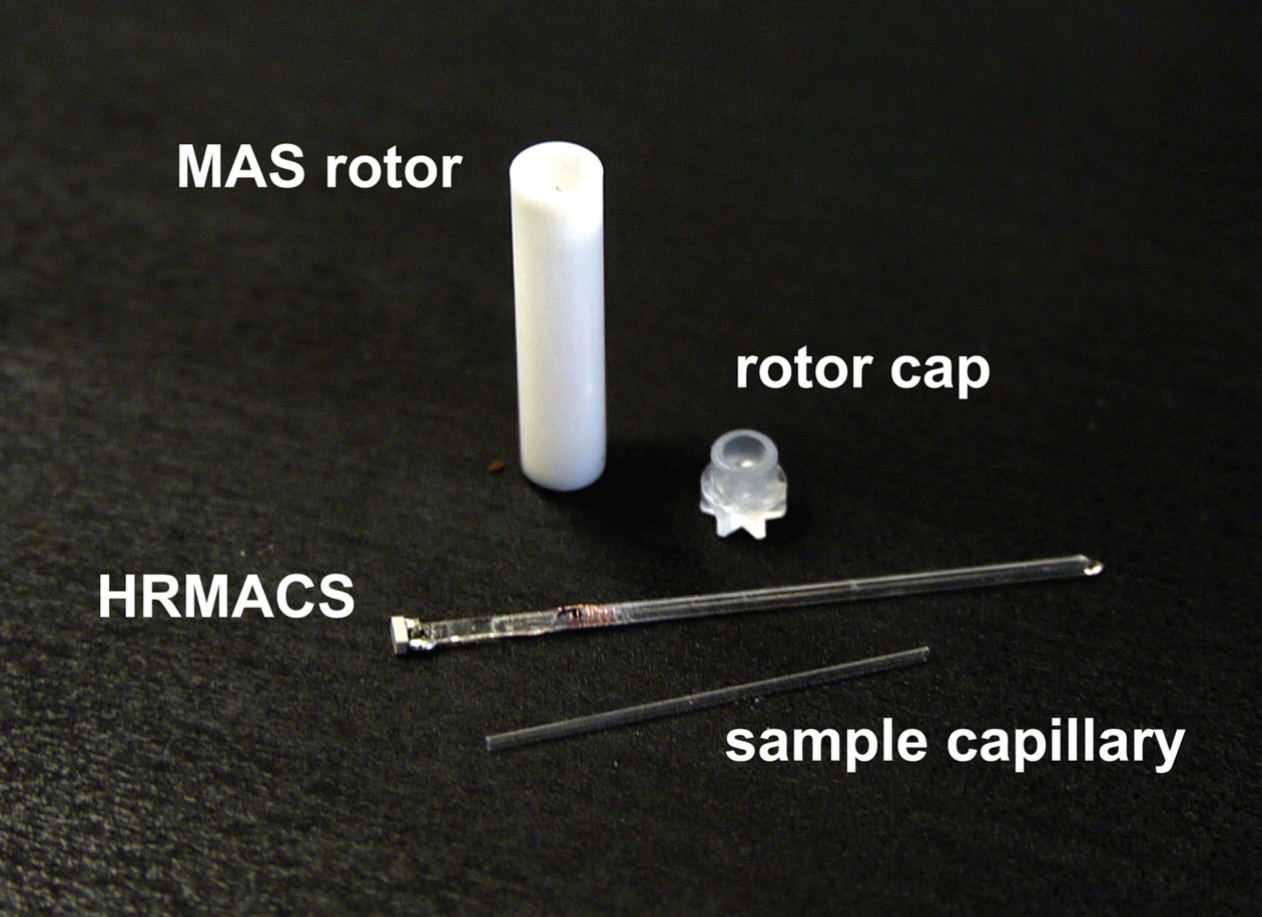
**A photo-illustration of the sample-exchange HR-MACS setup used in this study**.

A single sample-exchangeable HR-MACS resonator (Figure 1) was used in the study for data acquisition. The resonator was constructed by manually winding a 2 mm long (9-turn solenoid), using 30-μm o.d. round copper wire around a 840/600-μm (outer/inner diameter) quartz capillary. The solenoid was then fixed in place with a thin layer of cyanoacrylate glue. A non-magnetic 2.2 pF capacitor (American Technical Ceramics, US) was soldered to the coil leads and affixed to the end of the capillary with cyanoacrylate glue. The resonator had a resonance frequency of 508 MHz with an unloaded coil quality factor, *Q* = 26. The resonator was secured in a Kel-F insert which fits tightly inside a Bruker ZrO_2_ 4-mm rotor allowing for an easy sampling with a 550/400-μm quartz capillary.

### Sample-preparation

The sample preparation for HR-MACS was performed under a stereomicroscope. The *S. cervisiae* cells (ca. 300 × 10^6^ cells in 4 μl of D_2_O) were pipetted into a 550/400-μm capillary sealed at one end (with epoxy) using a micropipette equipped with a 20 μl GELoader® tip (Eppendorf, US). Very gentle centrifugation was applied for 2–5 s to remove air bubbles and ensure the cells displaced into the solenoid region. Within the coil detection region there are ca. 19 × 10^6^ cells. The top of the sample capillary was then sealed with hot paraffin wax to prevent leakage during sample spinning, and inserted into the HR-MACS resonator already fitted inside the MAS rotor. The entire sample-preparation procedure was restricted to no more than 2 min to minimize the chances of sample tampering and degradation.

### ^1^H HR-MACS NMR spectroscopy

^1^H NMR experiments were performed on a widebore 11.7 T magnet equipped with a Bruker Avance II Spectrometer operating at 499.13 MHz with a standard Bruker 4-mm HX CP-MAS probe (with a HR-MACS resonator placed inside the probe). The MAS frequency was set using the manual settings of a standard Bruker MAS spin controller to 300 ± 1 Hz. The *B*_0_ shimming was performed on a sucrose-D_2_O sample under slow MAS and re-checked every five samples. The 90°-pulse for HR-MACS was 5.3 μs at 2 W of applied radio-frequency amplitude. An 8-step 2D-PASS sequence was used (Antzutkin et al., 1995), as previously demonstrated with the addition of water-suppression (Wong et al., 2010, 2013), to acquire high-resolution sideband- and water-free spectra for all samples. ^1^H PASS NMR spectra were acquired with 8 *t*_1_ increments with each consisting of 96 co-added scans for 20 k data points over a spectral width of 20 ppm. A 1 s recycle delay was used resulting in a 26-min experiment time. The experiments were performed under sample temperature regulation at 10°C. ^1^H chemical shifts were internally referenced to the alanine—CH_3_—doublet at δ = 1.47 ppm. For each group, ^1^H NMR spectra of five replicate samples were recorded to ensure reproducibility and reliability of the spectral data for interpretation.

### Multivariate data analysis

To reduce the complexity of the NMR data for the subsequent multivariate analysis, the spectra were reduced to 0.005 ppm-wide buckets over the spectral region between 0.87 and 9.00 ppm, with exclusion of the water region (4.7–5.1 ppm), and normalized by the total sum of intensities using MestReNova v8.1.4. Principal component analysis (PCA) and orthogonal partial least-square discriminant analysis (OPLS-DA) were applied to check the data homogeneity and identify the latent patterns and biomarkers using SIMCA-P 13 (Umetrics, Umea, Sweden). Variable data were centered prior to PCA and OPLS-DA.

## Results and discussion

### ^1^H HR-MACS NMR spectra

**Figure 2:**
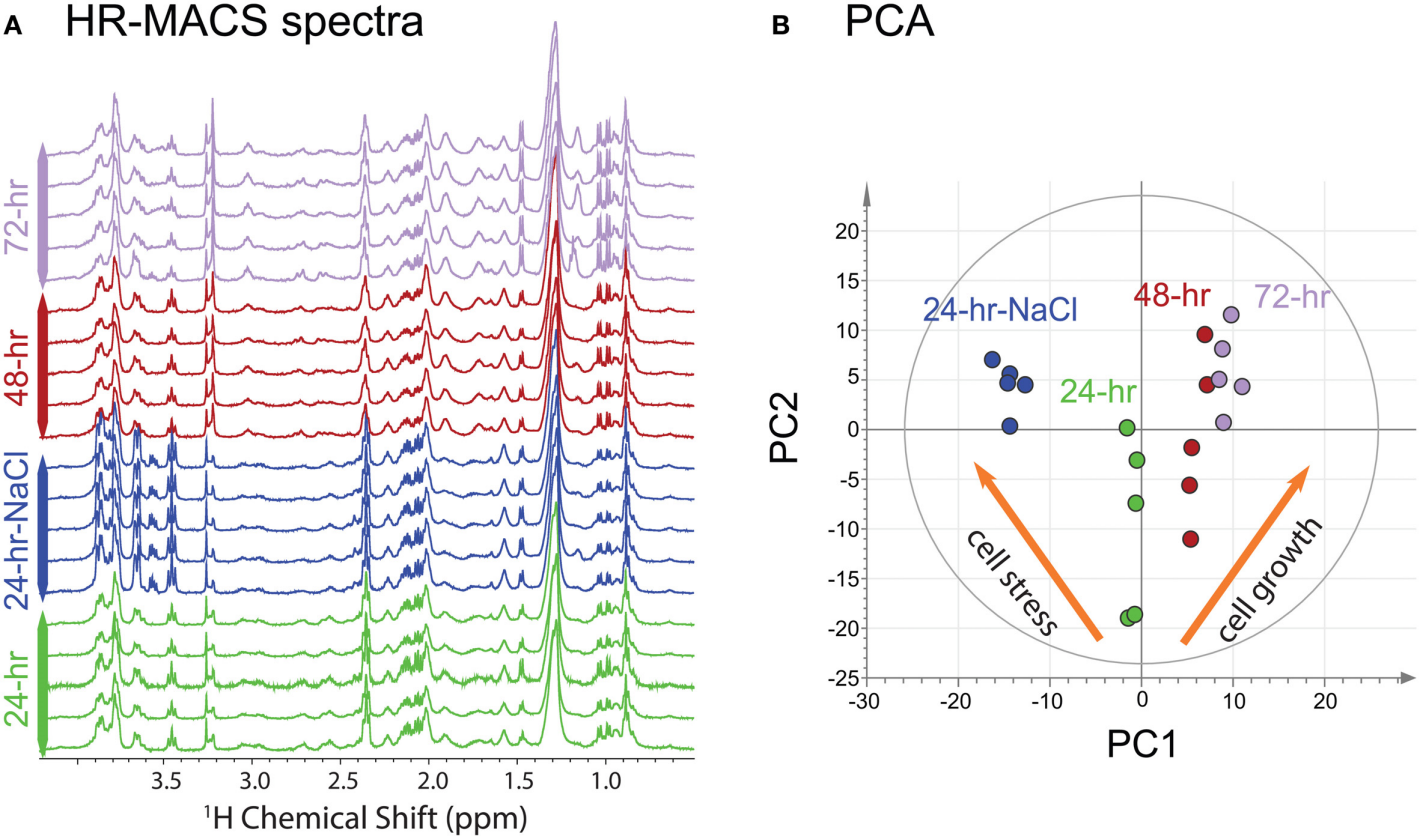
**(A)** ^1^H HR-MACS NMR spectra of the four different cell groups. **(B)** PCA score plot of all NMR datasets showing the quality of the sub-spectra [*R*^2^X(cum) = 0.793; *Q*^2^(cum) = 0.737). The inserted orange narrows show the different metabolic patterns for the two studies.

The ^1^H HR-MACS NMR spectra for all cell groups in this study (cell growth and osmotic study) are shown in Figure 2A. The overall spectral profiles and the resolution are consistent with the HR-MAS study using 30 μl volumes and ca. 75 million cells (Palomino-Schätzlein et al., 2013). Despite using a CP-MAS rather than a HR-MAS probe with field-locking capabilities, we nonetheless obtained excellent quality as previously shown (Wong et al., 2013), but better performance is expected with a HR-MAS probe. The use of the PASS experiment under slow MAS (300 Hz), which consists a train of 180-degree refocussing pulses within one rotor period (3.3 ms), provides a T_2_-filter similar to the T_2_-edited CPMG experiments for suppressing signals from large molecules that may mask the metabolite signals and distort the baselines. All cell groups were found to have high glucose (δ = 3.5–4.0 ppm) contents, which is attributed to the use of a glucose-rich growth medium (YPD) for the cultivation of cells. The observable fine *J*-splittings in many resonances are measureable from the spectra. For example, the distinct doublets of valine at 0.98 and 1.04 ppm (*J* = 7.3 Hz), and the triplet of α-glucose at 3.45 ppm (*J* = 9.6 Hz) are apparent in all HR-MACS spectra. The excellent spectral resolution allows extraction of this vital second parameter—in addition to chemical shift—permitting greater precision in peak assignments. A total of 22 metabolites have been identified from the two studies and are summarized in Table S1 of the supporting information (SI). The capability of such rich-profiling from a sub-microliter sample volume (250 nl) is owed to the fact that HR-MACS offers a 4.8-fold sensitivity enhancement in signal-to-noise (SNR) per-unit-mass compared to the coupled HR-MAS probe. This enhancement factor has been calculated based on *B^HRMACS^*_1_/*B^HRMAS^*_1_ at a given radio frequency input power (Hoult, 2000), where the *B*_1_ field can be determined from a standard nutation experiment. We found that with an estimated 19 million cells in 250 nl, we obtained an average SNR (using the 2.5–2.0 ppm spectral region) of 120 ± 10 for a 26-min experimental time. Such high SNR should allow for further reductions in sample size, or in signal-averaging for samples which are prone to rapid decay. Although, there are multiple factors that contribute to the SNR using the inductively-coupled HR-MACS resonator such as coil volume, sampling spin and voltage noise (more detailed descriptions can be found in SI), using our measured SNR and a value of 10:1 (SNR) as the minimum required for metabolite analysis, these results would indicate that with this HR-MACS spectra should be obtainable from ca. 2 million cells under the same experimental conditions.

Visual inspection of the spectra in Figure 2A allows discrimination of the spectral differences among the different cell groups. For example, higher intensities are found in the region between 3.5–4.0 ppm (glycerol and α-glucose) in the 24-h-NaCl group (cells under osmotic stress); larger signals at 1.0 ppm (valine) and at 3.2 ppm (phosphorylcholine and glycerophosphocholine) are also observed in the 72-h cells. Upon a more careful inspection, additional metabolites (choline, creatine, tyrosine, and phenylalanine) are identified in the aging cells as compared to stressed cells (see Table S1). An unsupervised PCA score plot is shown in Figure 2B. It reveals partitioning into four discrete clusters corresponding to the different cell groups. Interestingly, the plot also reveals a clear distinction between the two independent studies, with the corresponding cells responding in an opposite fashion along the first principal component (PC1). The PCA loading plot (Figure S1b), provides further evidence for metabolite differences between the two studies. As the cells age both phosphorylcholine and glycerophosphocholine increase; whereas, the metabolites glycerol, glucose and glutamine show the largest response to the osmatic shock induced by the additional NaCl.

### Metabolic profiling of *S. cervisiae* WT cells under osmotic stress

**Figure 3:**
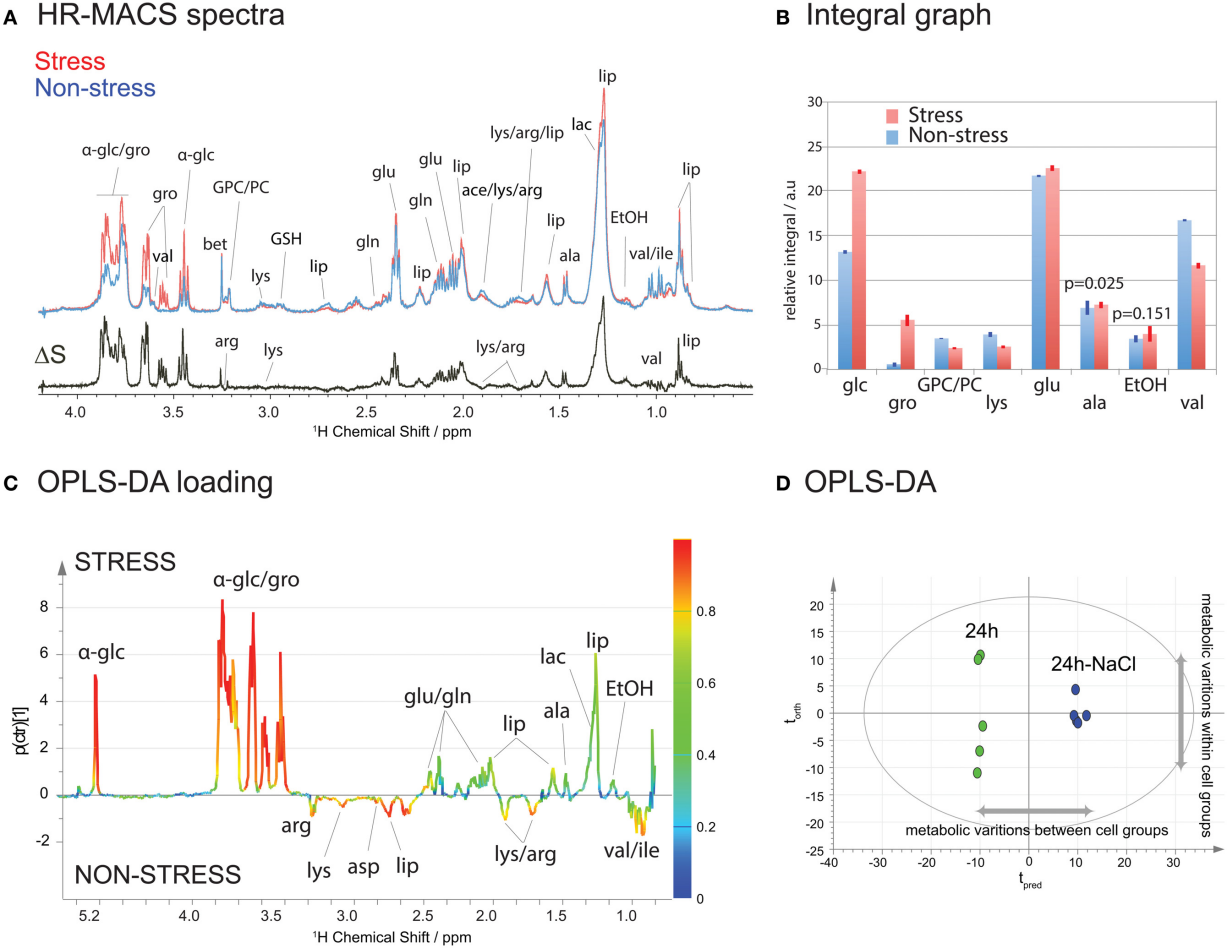
**^1^H HR-MACS NMR study of stressed cells. (A)** Spectral comparison of averaged spectra (*n* = 5): red corresponds to stressed cells and blue to non-stressed cells. The ΔS spectrum is the spectral difference between stressed and non-stressed cells, where the positive peaks correspond to the increased in metabolite contents in stressed cell, and the negative peaks in the non-stressed cells. The assignments including the abbreviations are listed in Table S1 in SI. **(B)** Bar-graph representation of the relative integral in arbitrary units for different metabolites. The results are reported with the mean values and the standard deviation as error bars. The *p*-value for each metabolite comparison is <0.001 with the exception of alanine and ethanol, which are stated in the bar-graph. Detailed information can be found in Table S2. **(C)** OPLS-DA score and **(D)** loadings plots with *Q*^2^ = 0.987, *R*^2^Y(cum) = 0.995 and *R*^2^X(cum) = 0.965.

Figure 3A shows a HR-MACS spectral comparison between the non-stressed and stressed cell groups. Both are average spectra, constructed using 5 replicate samples for each group. The difference spectrum, ΔS, consists only of the metabolites which vary under a 60-min osmotic shock; positive signals indicate an increase, and negative signals a decrease in a given metabolite upon osmotic shock. Substantial increases are found for both glucose and glycerol, and a decrease in a few amino acids (lysine, arginine, and valine) is observed. These changes have been quantified (Table S2 of SI) and are shown graphically in the spectral integral analysis of Figure 3B. For a more accurate metabolic analysis of the latent patterns, the acquired spectra were subjected to a supervised statistical analysis using OPLS-DA. The score plot (Figure 3C) displays a clear discrimination between the two groups. Interestingly, it shows a greater metabolic variation (i.e., along the orthogonal component *t*_orth_) among the non-stressed cells agreeing with the observed scatter pattern in the unsupervised PCA (Figure 2B). The corresponding loadings plot (Figure 3D) illustrates the metabolites associated with the separation in the score plot. As with the ΔS spectrum for Figure 3A, the positive signals are associated with the metabolites in higher concentration for the stressed cells and the negative peaks represent higher concentration in the non-stressed cells. The color scale provides a measure of the correlation of that spectral region's (i.e., metabolites') variation to the multivariate discrimination. Similar to the spectral comparison in Figures 3A,B, significant productions of glucose and glycerol metabolites are evident in the stressed cells (in agreement with the PCA loading plot Figure S2). This is inline with studies which have found that glycerol plays an osmoregulatory role in yeast cells (Tamas et al., 1999; Costenoble et al., 2000; Dihazi et al., 2004), and is produced when the yeast cells are subject to osmotic shock. The OPLS-DA analysis also reveals the latent metabolic changes; decreases in select amino acids (lysine, aspartate, arginine, and valine) may suggest an altered glucose metabolism that involves pyruvate production. Unfortunately, the chemical shift signature (a singlet at 2.36 ppm) for pyruvate is not readily identifiable in the spectra. The metabolic results obtained from ^1^H HR-MACS are coherent with the previous ^1^H HR-MAS study (Palomino-Schätzlein et al., 2013) using a large sample volume with greater number of cells, validating the used of HR-MACS.

### Metabolic profiling of *S. cervisiae* WT cells at three different stages of cell growth

**Figure 4:**
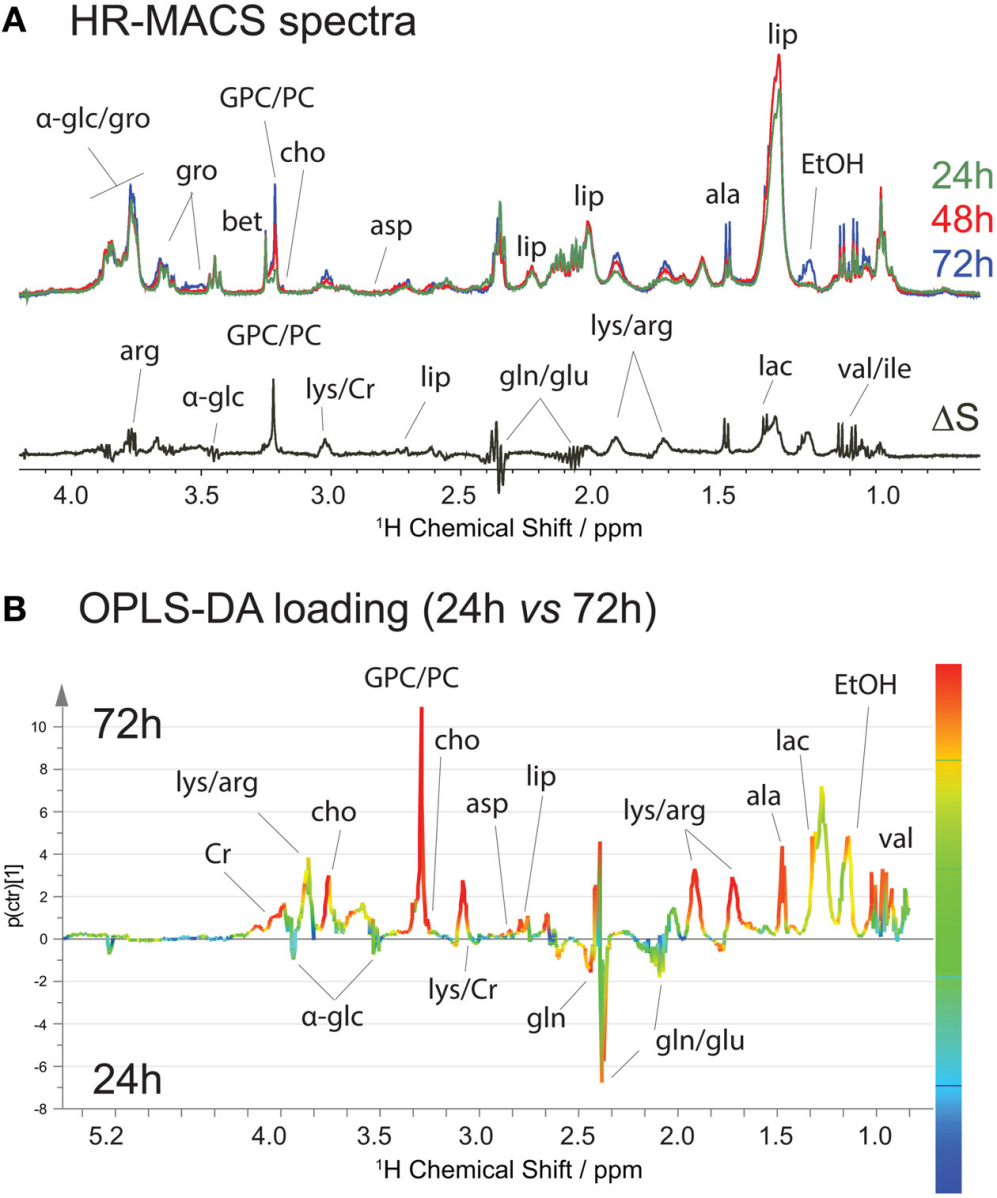
**^1^H HR-MACS NMR study of cell growths. (A)** Spectral comparison composed of averaged spectra (with *n* = 5): green corresponds to cell growth at 24-h; red at 48-h; and blue at 72-h. The ΔS spectrum is the spectral difference between 72 and 24-h cells. The positive peaks correspond to the increased in metabolite contents in 72-h cells, and the negative peaks in the 24-h cells. Both the assignments and abbreviations are listed in Table S1. **(B)** OPLS-DA loading with *Q*^2^ = 0.986, *R*^2^Y(cum) = 0.986 and *R*^2^X(cum) = 0.857.

We also monitored the metabolic profiles at three different stages of cell growth: 24-, 48- and 72-h. Typically, the *S. cervisiae* cells are in the exponential growth phase between 24- and 48-h interval, and reach a steady stage at 48-h. At 72-h, the cells may begin to breakdown the nutrients. Figure 4A shows the average spectral comparison (*n* = 5) between the different stages, with a differential ΔS spectrum of 24- and 72-h cell groups; the positive signals indicate an increase, and negative peaks a decrease of a given metabolite in the 72-h cell groups. Figure 4A clearly exhibits a metabolic spectral profile that is different from that for the stressed cells, suggesting that the metabolic variations are different in both studies. Increases in the metabolite content of ethanol, lipid, lactate, alanine, arginine, creatine, phosphorylcholine, and glycerophosphocholine are accompanied by decreases mainly in glutamate and glutamine. These changes are also found in the OPLS-DA analysis (Figure 4B), but with additional quantification of their contribution to the variation. The production of ethanol and depletion of glucose are attributed to the fact that the *S. cervisiae* cells are cultivated in a glucose-rich medium, resulting in initiation of the fermentation process (utilizing glucose and producing ethanol) and repression of respiration. The biosynthesis of the phosphocholine derivatives, the major phospholipid component of eukaryotic cell membranes, as well as choline are derived from its synthesis and catabolism (phosphatidylcholine metabolism) contributing to cell growth (Howe and McMaster, 2001).

It should be emphasized that good spectral quality data is vital for reliable metabolic identification and assessment, and acquisition of high quality data from small sample volumes is not a trivial task. It is often hindered by a lack of sensitivity and/or resolution, due to poor sample filling factor, or magnetic susceptibility gradients. The success of acquiring excellent spectral quality with HR-MACS is because it offers a near optimal filling factor and minimizes susceptibility-induced broadening by ensemble magic-angle spinning of the resonator with the sample. To our knowledge, there are no other analytical techniques capable of offering rich-metabolic analyses with such a small sample volume (250 nl) without damaging or interfering with the sample anatomy, including MS.

We would like to briefly discuss the benefits and drawbacks of HR-MACS for small-volume analysis of cells. HR-MACS offers a simple and cost-effective approach to high-quality μNMR analysis (materials cost for one coil < €20). The combination of slow sample spinning (300 Hz) and small sample diameter (i.e., 400 μm i.d. HR-MACS) minimize the centrifugal forces exerted upon the cells under rotation, thus making it amenable to analysis of more fragile cells. Another advantage is reduction in numbers of required cells; a 10-fold reduction in total cell quantity from that typically used (i.e., 20 samples of 100 × 10^6^ cells) should be readily achievable. ^1^H-detected multiple-resonance NMR experiments are also compatible with the HR-MACS approach (Aguiar et al., 2011), permitting the use of HSQC (and related) experiments for in-cell NMR spectroscopic investigations of the bioactivities inside the living cells (Li, 2006; Maldonado et al., 2011). The ability to analyze nano-scale volumes of cells opens new opportunities for coupling with cell-sorting microfluidic devices for a potent NMR cell screening. Microfluidic ^1^H NMR has recently emerged as a micro-NMR diagnostic device by differentiating the spin relaxation mechanisms between healthy and unhealthy cells; however, it yields minimal spectral information due to the poor resolution (Haun et al., 2011).

Although the cost is low, the manufacture of the small and delicate HR-MACS resonator can be challenging. Replacing the manual fabrication of HR-MACS with an automated system may make this technique more widespread (Malba et al., 2003; Badilita et al., 2012). The very-low absolute metabolite contents—despite the fact that HR-MACS enhances the detection sensitivity—hinder their detection, without resorting to greater signal averaging and thus, longer experiment times. Coupling with other sensitivity enhancement techniques (i.e., nuclear polarization) would shorten the experimental times. Another option to further improve the sensitivity is to build a standalone μHR-MAS probe (without HR-MACS) for eliminating the any noise contribution from the HR-MACS resonator itself; however, all above options would involve complex instrumentation developments.

## Concluding remarks

In this short report, we have demonstrated, for the first time, the ^1^H NMR metabolic profiling of a small quantity of whole cells on a nanolitre volume. The ^1^H NMR spectra acquired with HR-MACS are of excellent-quality and exhibit high-reproducibility facilitating a rich-metabolic profiling. The two cellular studies demonstrated here illustrate the ability of ^1^H HR-MACS coupled with statistical methods to provide high precision metabolic analyses of *S. cervisiae* cells in different conditions. Under an osmotic shock, the bio-production of glucose and glycerol metabolites and depleting glutamate and glutamine in *S. cervisiae* cells is evident. Significant increases in phosphatidylcholine and glycerophosphocholine are found in relation to cell growth and the production of ethanol via the fermentation process in the aging cells is also evident. The metabolic results found in the stressed cells using HR-MACS are in agreement with the previous HR-MAS study, validating the use of HR-MACS for small cell quantity study. ^1^H HR-MACS NMR spectroscopy opens new possibilities for high-precision investigation of small-volume sample, and may widen the scope of yeast metabolome and other bacterial cells, and the limited number of neuron cells in organisms. The ability to acquire ^1^H NMR spectra of such small volumes puts HR-MACS closer to the scales utilized for microfluidic-based cell sorting and manipulation techniques, facilitating their coupling for a potent micro-scale cell NMR sample screening pipeline.

### Conflict of interest statement

The authors declare that the research was conducted in the absence of any commercial or financial relationships that could be construed as a potential conflict of interest.

## References

[B1] Aguiar P. M. Jacquinot J.-F. Sakellariou D. (2011). A convenient, high-sensitivity approach to multiple-resonance NMR at nanolitre volumes with inductively-coupled micro-coils. Chem. Commun. 47, 2119–2121 10.1039/c0cc04607h 21180739

[B2] Antzutkin O. N. Shekar S. C. Levitt M. H. (1995). Two-dimensional sideband separation in magic-angle-spinning NMR. J. Magn. Reson. A 115, 7–19 10.1006/jmra.1995.11427834323

[B3] Badilita V. Fassbender B. Kratt K. Wong A. Bonhomme C. Sakellariou D. (2012). Microfabricated inserts for magic angle coil spinning (MACS) wireless NMR spectroscopy. PLoS ONE 7:e42848 10.1371/journal.pone.0042848 22936994 PMC3423418

[B4] Beckonert O. Coen M. Keun H. C. Wanga Y. Ebbels T. M. D. Holmes E. (2010). High-resolution magic-angle-spinning NMR spectroscopy for metabolic profiling of intact tissues. Nat. Protoc. 5, 1019–1032 10.1038/nprot.2010.45 20539278

[B5] Blaise B. J. Giacomotto J. Elena B. Dumas M.-E. Toulhoat P. Ségalat L. (2007). Metabotyping of *Caenorhabditis elegans* reveals latent phenotypes. Proc. Natl. Acad. Sci. U.S.A. 104, 19808–19812 10.1073/pnas.0707393104 18077412 PMC2148380

[B6] Chauton M. S. Optun O. I. Bathen T. F. Volent Z. Gribbestand I. S. Johnsen G. (2003a). HR MAS ^1^H NMR spectroscopy analysis of marine microalgal cells. Mar. Echo. Prog. Ser. 256, 57–62 10.3354/meps256057

[B7] Chauton M. S. Størseth T. R. Johnsen G. (2003b). High-resolution magic angle spinning 1H NMR analysis of whole cells of *Thalassiosira pseudonana* (*Bacillariophyceae*): broad range analysis of metabolic composition and nutritional value. J. Appl. Phycol. 15, 533–542 10.1023/B:JAPH.0000004355.11837.1d

[B8] Costenoble R. Valadi H. Gustafsson L. Niklasson C. Franzen C. J. (2000). Microaerobic glycerol formation in *Saccharomyces cerevisiae*. Yeast 16, 1483–1495 10.1002/1097-0061(200012)16:16<1483::AID-YEA642>3.0.CO;2-K 11113971

[B9] Dihazi H. Kessler R. Eschrich K. (2004). High osmolarity glycerol (HOG) pathway-induced phosphorylation and activation of 6- phosphofructo-2-kinase are essential for glycerol accumulation and yeast cell proliferation under hyperosmotic stress. J. Biol. Chem. 279, 23961–23968 10.1074/jbc.M312974200 15037628

[B10] Dunn W. B. Ellis D. I. (2005). Metabolomics: current analytical platforms and methodologies. Trends Anal. Chem. 24, 285–294 10.1016/j.trac.2004.11.02122212615

[B11] Gudlavalleti S. K. Szymanski C. M. Jarrell H. C. Stephens D. S. (2006). *In vivo* determination of Neisseria meningitidis serogroup A capsular polysaccharide by whole cell high-resolution magic angle spinning NMR spectroscopy. Carbohydr. Res. 341, 557–562 10.1016/j.carres.2005.11.036 16406275

[B12] Haun J. B. Castro C. M. Wang R. Peterson V. M. Marinelli B. S. Lee H. (2011). Micro-NMR for rapid molecular analysis of human tumor samples. Sci. Transl. Med. 3, 71ra16 10.1126/scitranslmed.3002048 21346169 PMC3086073

[B13] Himmelreich U. Somorjai R. L. Dolenko B. Lee O. C. Daniel H.-M. Murray R. (2003). Rapid identification of Candida species by using nuclear magnetic resonance spectroscopy and a statistical classification strategy. Appl. Environ. Microbiol. 69, 4566–4574 10.1128/AEM.69.8.4566-4574.2003 12902244 PMC169103

[B14] Hoult D. I. (2000). The principle of reciprocity in signal strength calculations - a mathematical guide. Concept Magn. Reson. 12, 173–187 10.1002/1099-0534(2000)12:4<173::AID-CMR1>3.0.CO;2-Q

[B15] Howe A. G. McMaster C. R. (2001). Regulation of vesicle trafficking, transcription, and meiosis: lessons learned from yeast regarding the disparate biologies of phosphatidylcholine. Biochim. Biophys. Acta. 1534, 65–77 10.1016/S1388-1981(01)00181-0 11786293

[B16] Li W. (2006). Multidimensional HRMAS NMR: a platform for *in vivo* studies using intact bacterial cells. Analyst 131, 777–781 10.1039/b605110c 16874945

[B17] Lindon J. C. Beckonert O. P. Holmes E. Nicholson J. K. (2009). High-resolution magic angle spinning NMR spectroscopy: application to biomedical studies. Prog. Nucl. Magn. Reson. 55, 79–100 10.1016/j.pnmrs.2008.11.004

[B18] Malba V. Maxwell R. Evans L. B. Bernhardt A. E. Cosman M. Yan K. (2003). Laser-lathe lithography - a novel method for manufacturing nuclear magnetic resonance microcoils. Biomed. Microdevices 5, 21–27 10.1023/A:1024407231584

[B19] Maldonado A. Y. Burz D. S. Shekhtman A. (2011). In-cell NMR spectroscopy. Prog. Nucl. Magn. Reson. Spectrosc. 59, 197–212 10.1016/j.pnmrs.2010.11.002 21920217 PMC3175053

[B20] Palomino-Schätzlein M. Molina-Navarro M. M. Tormos-Pérez M. Rodríguez-Navarro S. Pineda-Lucena A. (2013). Optimised protocols for the metabolic profiling of *S. cerevisiae* by ^1^H-NMR and HR-MAS spectroscopy. Anal. Bioanal. Chem. 405, 8431–8441 10.1007/s00216-013-7271-9 23942588

[B21] Reo N. V. (2002). NMR-based metabolomics. Drug Chem. Toxicol. 25, 375–382 10.1081/DCT-120014789 12378948

[B22] Righi V. Apidianakis Y. Mintzopoulos D. Astrakas L. Rahme L. G. Tzika A. A. (2010). *In vivo* high-resolution magic angle spinning magnetic resonance spectroscopy of Drosophila melanogaster at 14.1 T shows trauma in aging and in innate immune-deficiency is linked to reduced insulin signaling. Int. J. Mol. Med. 26, 175–184 10.3892/ijmm_00000450 20596596 PMC3722717

[B23] Righi V. Constantinou C. Kearwani M. Rahme L. G. Tzika A. A. (2013). Live-cell high resolution magic angle spinning magnetic resonance spectroscopy for *in vivo* analysis of *Pseudomonas aeruginosa* metabolomics. Biomed. Rep. 1, 707–712 10.3892/br.2013.148 24649014 PMC3917020

[B24] Sakellariou D. Le Goff G. Jacquinot J.-F. (2007). High-resolution, high-sensitivity NMR of nanolitre anisotropic samples by coil spinning. Nature 447, 694–698 10.1038/nature05897 17554303

[B25] Sitter B. Bateh T. F. Tessem M.-B. Gribbestad I. S. (2009). High-resolution magic angle spinning (HR MAS) MR spectroscopy in metabolic characterization of human cancer. Prog. Nucl. Magn. Reson. 54, 239–254 10.1016/j.pnmrs.2008.10.001

[B26] Tamas M. J. Luyten K. Sutherland F. C. Hernandez A. Albertyn J. Valadi H. (1999). Fps1p controls the accumulation and release of the compatible solute glycerol in yeast osmoregulation. Mol. Microbiol. 31, 1087–1104 10.1046/j.1365-2958.1999.01248.x 10096077

[B27] Wong A. Aguiar P. M. Sakellariou D. (2010). Slow magic-angle coil spinning: a high-sensitivity and high-resolution NMR strategy for microscopic biological specimens. Magn. Reson. Med. 63, 269–274 10.1002/mrm.22231 20099320

[B28] Wong A. Jiménez B. Li X. Holmes E. Nicholson J. K. Lindon J. C. (2012). Evaluation of high resolution magic-angle coil spinning NMR spectroscopy for metabolic profiling of nanoliter tissue biopsies. Anal. Chem. 84, 3843–3848 10.1021/ac300153k 22449140

[B29] Wong A. Li X. Sakellariou D. (2013). Refined magic-angle coil spinning resonator for nanoliter NMR spectroscopy: enhanced spectral resolution. Anal. Chem. 85, 2021–2026 10.1021/ac400188b 23343461

