# Supplementary material for: 1H high resolution magic-angle coil spinning (HR-MACS) μNMR metabolic profiling of whole Saccharomyces cervisiae cells: a demonstrative study
Source: Front Chem. 2014 Jun 12;2:38. doi: 10.3389/fchem.2014.00038 (PMC4053607; doi:10.3389/fchem.2014.00038)
Supplement: Supplementary file 1 [file DataSheet1.PDF]

Supporting Information

For

^{1}H High Resolution Magic-Angle Coil Spinning (HR-MACS) µNMR Metabolic Profiling of whole *Saccharomyces cervisiae* cells*: A Demonstrative Study*

Alan Wong[1]*, Céline Boutin[1], Pedro M. Aguiar[2]

[1] CEA Saclay, DSM, IRAMIS, UMR CEA/CNRS 3299 – NIMBE, Laboratoire Structure et Dynamique par Résonance Magnétique, F-91191, Gif-sur-Yvette Cedex, France.

[2] Department of Chemistry, University of York, Heslington, York, YO10 5DD, United Kingdom

Table of Contents

Table S1 : Peak Assignments and J-splittings S2

Table S2 : Metabolite integrals of stressed and non-stressed cells S3

Figure S1 : PCA score and Loading plots S4

Relation of SNR to coil volume and detectable nuclear spin S5

References S6

Table S1 Peak assignment of the identified metabolites found in the wild-type strains of *S. cervisiae* from both the aged cell group and the stressed cell group.

^{1}H shift / ppm (multiplicity)[a]	metabolite	abbreviation	observable J-splitting / Hz
0.84(t)	lipid CH3	Lip	
0.88(t)	lipid CH3	Lip	~7.0
0.93(t)	isoleucine	Ile	–
0.98(d)	valine	Val	7.3
1.01(m)	isoleucine	Ile	–
1.04(d)	valine	Val	7.3
1.19(t)	Ethanol	EtOH	7.4
1.27(m)	lipid $-(CH_2)_n-$	Lip	–
1.32(d)	lactate	Lac	6.4
1.47(d)	alanine	Ala	7.6
1.57(m)	lipid $-(CH_2)_nCO$	Lip	–
1.65(m)	arginine	Arg	–
1.72(m)	lysine/lipid $CH_2CH_2C{=}C$	Lys/Lip	–
1.90(m)	arginine/lysine	Arg/Lys	–
1.93(s)	acetate	Ace	–
2.01(m)	lipid $CH_2C{=}C$	Lip	–
2.06(m)	glutamate	Glu	–
2.12(m)	glutamine	Gln	–
2.23(m)	lipid CH_2CO	Lip	–
2.36(t)	glutamate	Glu	–
2.45(m)	glutamine	Gln	–
2.56(m)	unknown		–
2.68(m)	aspartate	Asp	–
2.73(m)	lipid $C{=}CCH_2C{=}C$	Lip	–
2.76(m)	lipid $C{=}CCH_2C{=}C$	Lip	–
2.81(dd)	aspartate	Asp	~17.0, ~3.2
2.95(m)	Glutathione	GSH	–
3.04(m)	creatine (in 48- and 72-hr)	Cr	–
3.04(t)	lysine	Lys	~8.0
3.19(s)	choline (in 48- and 72-hr)	Cho	–
3.21(s)	PC	PC	–
3.22(s)	GPC	GPC	–
3.24(m)	arginine	Arg	–
3.26(2)	betaine	Bet	–
3.45(t)	α-glucose	α-Glc	9.6
3.55(dd)	glycerol	Gro	11.8, 6.7
3.61(d)	valine	Val	4.6
3.64(dd)	glycerol	Gro	~10.3, ~2.7
3.68(m)	choline (lipid) (in 72-hr)	Cho	–
3.77(m)	α-glucose	α-Glc	–
3.83(m)	α-glucose/glycerol	α-Glc/Gro	–
3.95(m)	creatine (in OPLS-DA 72-hr)	Cr	–
5.19(d)	α-glucose	α-Glc	3.0
5.31(m)	UFA	UFA	–
6.88(d)	tyrosine (in 72-hr)	Tyr	7.3
7.18(d)	tyrosine (in 72-hr)	Tyr	7.0
7.32(m)	phenylalanine (in 72-hr)	Phe	–
7.37(m)	phenylalanine (in 72-hr)	Phe	–

UFA = unsaturated fatty acids; PC = phosphorylcholine; GPC = glycerophosphocholine

a) The assignments are based on previous assignments [1]. Abbreviations: s = singlet; d = doublet; dd = doublet of doublets; t = triplet; m = multiplet

Table S2. Normalized integration[a] of the metabolite resonances for the two different *S. cervisiae.groups: control cells and stressed cells*

metabolite	integral region	**Control** mean integral (n=5)	**24-hr** STD	**Stress** mean integral (n=5)	**24-hr-NaCl** STD	Δintegral (stressed – control)[b]	p-value[c]
α-Glu	5.202-5.177<br>3.482–3.420	13.19	0.18	22.23	0.22	+9.04	<0.001
Gro	3.577–3.523	0.60	0.22	5.57	0.61	+4.97	<0.001
GPC/PC	3.228–3.209	3.49	0.06	2.41	0.10	–1.08	<0.001
Lys	3.081–3.029	3.92	0.27	2.56	0.14	–1.36	<0.001
Glu	2.379–2.328	25.31	0.11	25.98	0.29	+0.68	<0.001
Ala	1.491–1.460	6.91	0.82	7.42	0.34	+0.51	0.025
EtOH	1.176–1.136	3.45	0.43	3.98	0.87	+0.53	0.151
Val	1.049–1.019<br>1.000–0.969	16.74	0.13	11.69	0.28	–5.05	<0.001

(a) Data are plotted in the bar-graph in Fig. 2b. (b) Positive indicates higher metabolite content in stressed cells, negative higher in the control cells. (c) Analyzed by the Student's *t-test* with a significant level at $p < 0.05$.

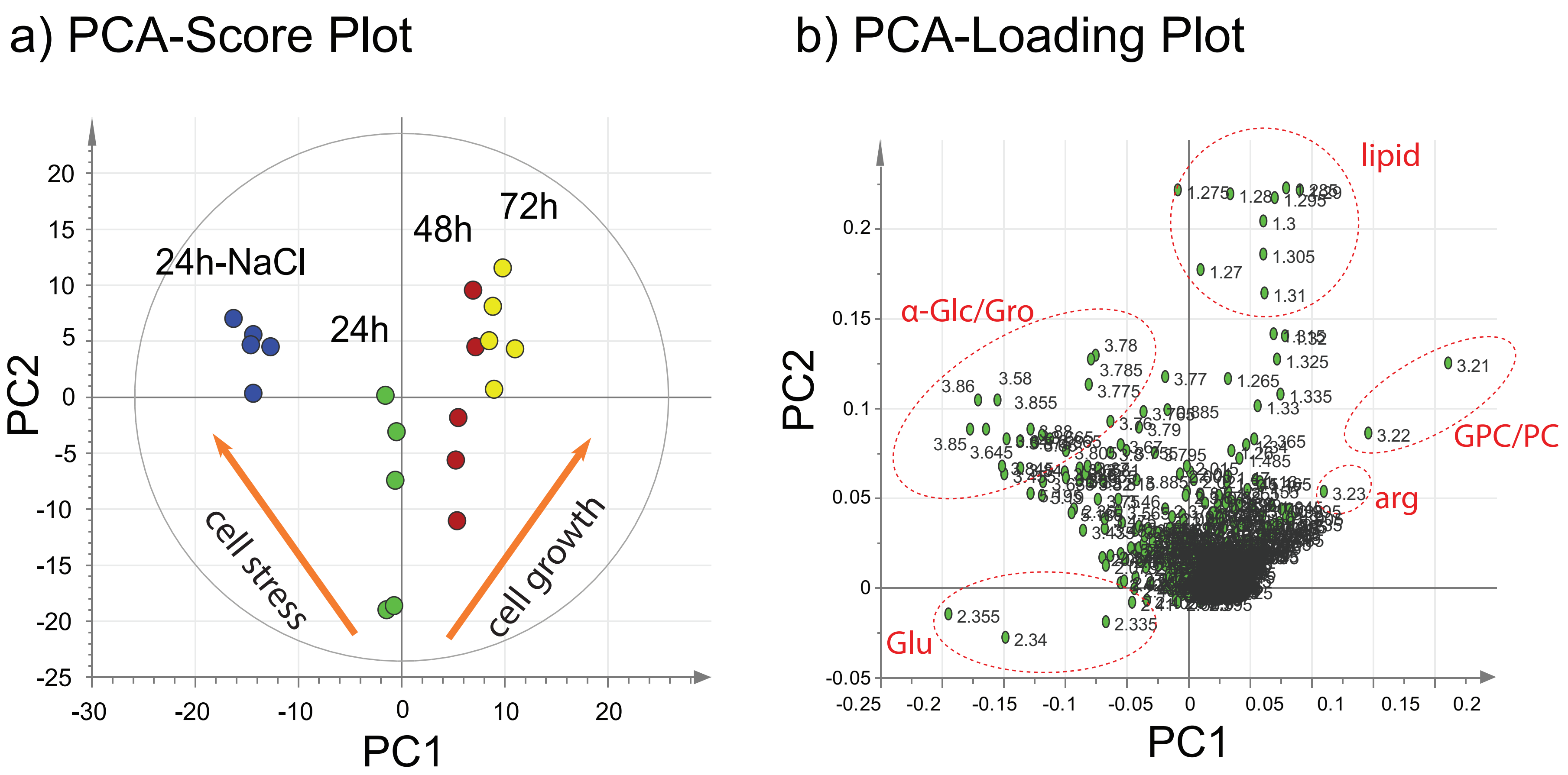

Figure S1. PCA score and its corresponding loading plot of all ^{1}H HR-MACS data acquired for the two studies, cell stress and cell growth. (R^2X(cum) = 0.793; Q^2(cum) = 0.737).

Signal-to-noise and Coil Volume

A complete formulation of the S/N (including sample and coil temperature, bandwidth effects etc. may be found in the literature) [2]. Assuming no change in the above contributions, we may work with a simplified expression for S/N, denoted by the following relation:

$$SNR = \frac{\text{peak signal}}{\text{RMS noise}} \propto \frac{\omega_0^2 (B_1/i) N_S}{V_{noise}} \qquad \text{(S1)}$$

where B_1/i is the radio-frequency field generated by the coil per unit current, N_S denotes the number of spins, V_{noise} is the voltage noise, and w_0 is the resonance frequency. Since $V_{noise} \propto \sqrt{r_{coil}}$ and the power dissipation, P, $P = r_{coil} i^2$, we may re-write equation S1 as the following:

$$SNR = \frac{\text{peak signal}}{\text{RMS noise}} \propto \omega_0^2 N_S \frac{B_1}{\sqrt{P}} \qquad \text{(S2)}$$

The value $B_1/\sqrt{P}$ varies with the volume and geometry of the solenoid. For a solenoid of given aspect ratio (ratio of length to diameter) we can take the definition of $B_1/\sqrt{P}$ given by Clark:[3]

$$\frac{B_1}{\sqrt{P}} \propto \sqrt{\frac{Q}{V_{coil}\omega_0}} \qquad \text{(S3)}$$

where, Q is the quality factor of the coil and V_{coil} is the volume of the coil and substitute into equation S2 to yield the following:

$$SNR = \frac{\text{peak signal}}{\text{RMS noise}} \propto \omega_0^{3/2} N_S \sqrt{\frac{Q}{V_{coil}}} \qquad \text{(S4)}$$

Equation S4 allows a direct assessment of the impact of the coil volume and number of spins present on the signal-to-noise obtainable. It should be noted that in the case of inductively-coupled micro-coils additional factors such as the regime of the coupling (i.e., under-, critically- or over-coupled) and the effects of resonance offset from the *exact* resonance frequency of the spins also play important roles and have been examined in detail elsewhere [3,4].

References:

1. (a) Nicholson, J. K.; Foxall, P. J. D.; Spraul, M.; Farrant, R. D.; Lindon, J. C., 750 MHz 1H and ^{1}H-^{13}C NMR spectroscopy of human blood plasma. *Anal. Chem.* **1995,** *67*, 793-811; (b) Palomino-Schätzlein, M.; Molina-Navarro, M. M.; Tormos-Pérez, M.; Rodríguez-Navarro, S.; Pineda-Lucena, A., Optimised protocols for the metabolic profiling of S. cerevisiae by ^{1}H-NMR and HR-MAS spectroscopy. *Anal. Bioanal. Chem.* **2013,** *405*, 8431-8441.

2. (a) Hoult, D. I.; Richards, R. E., The signal-to-noise ratio of the nuclear magnetic resonance experiment. *Journal of Magnetic Resonance* **1976,** *24*, 71-85; (b) Peck, T. L.; Magin, R. L.; Lauterbur, P. C., Design and Analysis of Microcoils for NMR Microscopy. *J. Magn. Reson. B* **1995,** *108*, 114-124.

3. Clark, W. G., Pulsed Nuclear Resonance Apparatus. *Rev. Sci. Instr.* **1964,** *35*, 316-333.

4. Jacquinot, J. F.; Sakellariou, D., NMR Signal Detection in Rotating Microcoil. *Concepts Magn. Reson. A* **2011,** *38*, 33-51.
